# User-centered design of a telehealth-enhanced hybrid cardiac rehabilitation program as hospital quality improvement

**DOI:** 10.21203/rs.3.rs-2475875/v1

**Published:** 2023-01-16

**Authors:** Andrea T. Duran, Adrianna Keener-DeNoia, Kimberly Stavrolakes, Adina Fraser, Luis V. Blanco, Emily Fleisch, Nicole Pieszchata, Diane Cannone, Charles Keys McKay, Emma Whittman, Donald Edmondson, Rachel C. Shelton, Nathalie Moise

**Affiliations:** Columbia University Irving Medical Center; Columbia University Irving Medical Center; New York Presbyterian Hospital; New York Presbyterian Hospital; Columbia University Irving Medical Center; New York Presbyterian Hospital; New York Presbyterian Hospital; Columbia University Irving Medical Center; Columbia University Irving Medical Center; Mailman School of Public Health; Columbia University Irving Medical Center; Mailman School of Public Health; Columbia University Irving Medical Center

**Keywords:** user-centered design, implementation science, cardiac rehabilitation, telemedicine

## Abstract

**Background:**

Innovative program designs and strategies are needed to support the widespread uptake of cardiac rehabilitation (CR) programs in the post-COVID 19 era. We combined user-centered design (UCD) and implementation science (ImS) principles to design a novel telehealth-enhanced hybrid (home and clinic-based) CR (THCR) program.

**Methods:**

As part of a New York Presbyterian Hospital (NYPH) quality improvement initiative (March 2020-February 2022), we designed a THCR program using an iterative 3 step UCD process informed by the Theoretical Domains Framework and Consolidated Framework for Implementation Research to: 1) identify user and contextual barriers to CR uptake (stakeholder interviews), 2) design an intervention prototype (design workshops and journey mapping), and 3) refine the prototype (usability testing). The process was optimized for usability and implementation outcomes.

**Results:**

Step 1: Semi-structured interviews with stakeholders (n = 9) at 3 geographically diverse academic medical centers revealed behavioral (e.g., self-efficacy, knowledge) and contextual (e.g., social distancing guidelines, physical space, staffing, reimbursement) barriers to uptake. Step 2: Design workshops (n = 20) and journey-mapping sessions (n = 3) with multi-disciplinary NYPH stakeholders (e.g., digital health team, CR clinicians, creative director) yielded a THCR prototype that leveraged NYPH’s investment in their remote patient monitoring (RPM) platform to optimize feasibility of home-based CR sessions. Step 3: Usability testing with CR clinicians (n = 2) administering and CR patients (n = 3) participating in home-based sessions revealed usability challenges (e.g., RPM devices/exercise equipment usability; Wi-Fi/Bluetooth connectivity/syncing; patient safety/knowledge and protocol flexibility). Design workshops (n = 24) and journey-mapping sessions (n = 3) yielded design solutions (e.g., onboarding sessions, safety surveys, fully supervised remote sessions) and a refined THCR prototype.

**Conclusion:**

Combining UCD and ImS methods while engaging multi-disciplinary stakeholders in an iterative process yielded a theory-informed telehealth-enhanced hybrid CR program targeting user and contextual barriers to real-world CR implementation. We provide a detailed summary of the process, and guidance for incorporating UCD and ImS methods in early-stage intervention development. THCR may shrink the evidence-to-practice gap in CR implementation. A future hybrid type I effectiveness-implementation trial will determine its feasibility, acceptability, and effectiveness.

## Background

Cardiac rehabilitation (CR)—which involves exercise training, patient education and health behavior modification in clinic- and/or home-based settings—is a Class I intervention, with a Level A recommendation for secondary prevention among cardiac patients.^1^ Despite the well-established effectiveness of CR,^2–4^ less than 27% of eligible cardiac patients participate in, and adhere to, CR programs in the United States. Undoubtedly, barriers to implementing *traditional* (i.e., clinic-based) CR have been identified (e.g., transportation, time/scheduling, motivation),^5–7^ with new implementation barriers emerging during COVID-19 (e.g., social distancing, patient fear).^8–12^ Due to the sustained low rates of participation in clinic-based CR programs, a 2019 scientific statement from expert organizations highlighted an urgent need to identify *nontraditional* models (e.g., home-based, alternative-site, hybrid) to improve CR participation and enhance widespread reach.^13^

Over the past two decades, nontraditional CR models have emerged as viable solutions due to their ability to overcome common patient-level barriers to clinic-based CR (e.g., transportation, time, etc.) and improve clinical and health-related quality of life outcomes with effect sizes similar to those of clinic-based CR.^13–15^ However, great variability in program design and barriers to uptake exist (distinct from clinic-based CR), including patient safety concerns, effective patient-provider communication, and inconsistent reimbursement of remote/home-based sessions.^13^ To overcome these barriers and ensure continuity of CR in the COVID-19 era, national and international scientific statements called for broader use of information and communication technologies (i.e., telehealth; e.g., websites, mobile phone applications, etc.) to deliver and integrate nontraditional CR into health care settings.^8,16^

Although widespread support for telehealth-enabled nontraditional CR exists, determining which design elements to include for a specific organizational/healthcare infrastructure and patient population remains unclear; partly because the multi-level factors that impede or support the successful implementation of such programs are not well defined. Further, the optimal design of a nontraditional CR program (e.g., frequency of home-based sessions, user-friendly telehealth devices) to improve patient and provider experiences and clinical outcomes in racial/ethnic and socioeconomically diverse settings has yet to be established.^17^ These myriad sources of uncertainty illustrate the need for guidance on how best to design nontraditional CR programs that address barriers and facilitators of uptake– not only at the patient level, but also at the provider and healthcare system levels. The challenges outlined make this gap (between the evidence of CR effectiveness and the uptake of CR programs in real-world settings) particularly ripe for an intervention development approach that combines user-centered design (UCD) principles, which utilize an iterative and highly stakeholder-engaged process to cocreate products that are directly responsive to the end user experience,^18,19^ and implementation science (ImS), which employs theoretical frameworks to target implementation barriers and elucidate key mediators/moderators of implementation outcomes (e.g., feasibility, acceptability),^20,21^ particularly at early stages of intervention development and refinement.^22,23^

Despite prior calls for combining UCD and ImS,^24–26^ few applied research examples demonstrating how to approach and operationalize this process, and fewer for CR, exist. Using a New York Presbyterian Hospital (NYPH) quality improvement (QI) project focused on improving CR uptake as a use case, we describe how we infused UCD principles and ImS methods to develop a telehealth-enhanced hybrid cardiac rehabilitation (THCR) program at Stage I of the NIH Stage Model of behavioral intervention development.^22,27^ To our knowledge, this is one of the first studies to describe a theory-informed, iterative approach to the design and implementation of a THCR program in a real-world academic medical setting, particularly at NIH Stage I intervention generation, refinement, and pilot testing. Our overarching goal is to improve the routine and equitable uptake of non-traditional CR programs at the patient, provider, and system level.

## Methods

### Evidence-based Practice and Context

Traditional CR is an evidence-based, standard of care, clinic-based program that includes patient assessment (medical history, functional capacity), exercise training (aerobic and strength), patient education/counseling (nutrition, psychosocial), and risk factor management (lipids, blood pressure, weight, diabetes mellitus, and smoking).^28^ The traditional CR model offers 24–36 sessions over 3–6 months at a facility (e.g., hospital, clinic, etc.). Each CR session is 60 minutes in duration, often occurs in group-based settings (e.g., 2–4 people), and is directly supervised in-person by a team of CR clinicians (e.g., physical therapist, nurse, exercise physiologist). Nontraditional CR targets the same core components as traditional CR but delivers CR sessions outside of the traditional clinic-based setting. Nontraditional CR programs can include home-based, virtual/telehealth/telemedicine-based, and/or community-based/unsupervised CR sessions or a combination of such sessions with at least one center-, facility-, clinic-based/supervised CR session (i.e., “hybrid” CR).^13,29,30^ Although nontraditional models have been encouraged, traditional clinic-based CR remains the most widely available across the U.S. and is established as a reimbursable service by Center for Medicare & Medicaid Services (CMS).^31,32^ The COVID19 pandemic halted many U.S. clinic-based CR services and created opportunities for rapid adoption of reimbursable telemedicine-enhanced programs.^10^

### Study Overview

**Figure 1:**
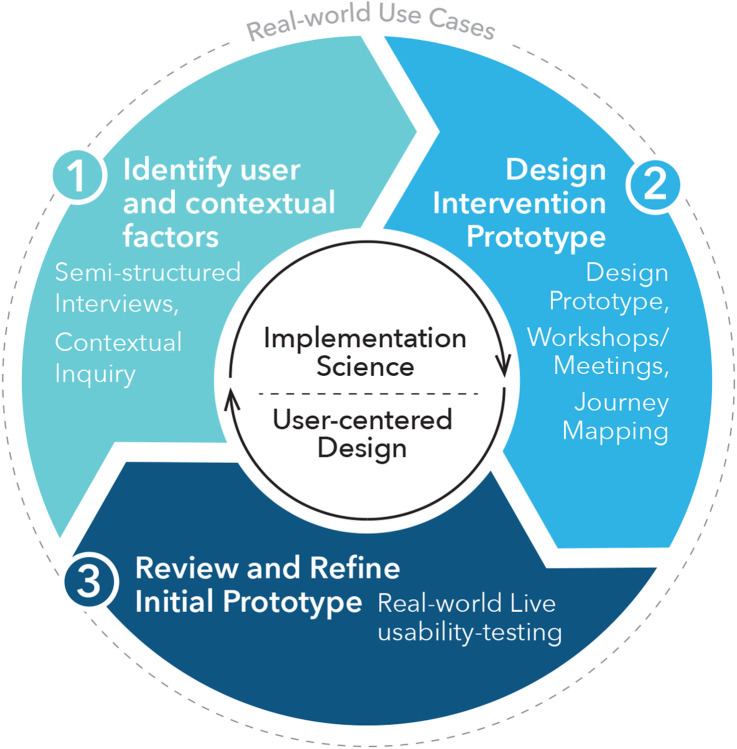
Overview of combined user-centered design and implementation science process to develop a nontraditional cardiac rehabilitation program. This figure outlines our iterative three step user-centered design (UCD) process to: 1) identify user and contextual factors (semi-structured interviews and contextual inquiry), 2) design an intervention prototype (design team meetings and journey mapping), and 3) review and refine the intervention prototype (real-world user-testing and feedback). To guide the UCD process, we employed the Theoretical Domains Framework and Consolidated Framework for Implementation Research.

As part of a NYPH quality improvement QI project (March 2020-February 2022), we sought to design a nontraditional CR program to offer patients in the post-COVID-19 era. We engaged in an iterative three-step UCD process to: 1) identify user and contextual factors that could influence uptake (using semi-structured interviews and contextual inquiry), 2) design an intervention prototype (through design team meetings and journey mapping), and 3) review and refine the intervention prototype (according to real-world user-testing and feedback). To guide the UCD process, we employed the Theoretical Domains Framework (TDF; 84 theoretical constructs within 14 domains [e.g., knowledge, skills, social/professional role])^33–35^ and Consolidated Framework for Implementation Research (CFIR; 39 constructs within five domains [e.g., inner setting, outer setting]).^36,37^ While there is considerable overlap in these theoretical implementation science frameworks, we leveraged the ways in which TDF addresses individual-level determinants (e.g., motivation and capability) and CFIR addresses system-level determinants (e.g., inner/organizational and outer/policy setting).^38^ All data collected as part of the NYPH QI project are covered by Human Subjects protocol IRB-AAAT2306. We applied the Standards for Quality Improvement Reporting Excellence Implementation Studies (SQUIRE2.0) when preparing this manuscript.^39^ Fig. 1 provides a conceptual model of the combined UCD and ImS process and methods. Table 1 provides an overview of the data collection methods, stakeholders, deliverables and targeted implementation (feasibility, appropriateness, acceptability) and usability outcomes (usefulness, usability) relevant to developing the nontraditional CR intervention.^26,40^ **Supplemental Table 1** provides descriptions and definitions of key UCD and ImS methods, frameworks, and terms included throughout the design process (e.g., CFIR, TDF, journey mapping, usability testing, contextual inquiry).

### Step 1 Methods: Identify user and contextual factors

From March to July 2020, we conducted semi-structured interviews with key stakeholders with expertise in CR, and in-depth interviews with providers from NYPH, as a form of contextual inquiry.^41^ The goal of this step was to understand barriers and facilitators of CR implementation that emerged during COVID-19, and acquire information necessary to optimize the adoption of CR in the era of remote clinical care.

#### Step 1A. Theory-informed Semi-structured Interviews

We conducted semi-structured, key informant interviews via video, telephone or in-person with researchers, clinicians, and administrators with expertise in CR (clinic- and/or home-based). We identified key stakeholders using a combination of academic literature review (e.g., scientific statements, home-based CR programs) and snowball sampling. Each interview was conducted in a rapid-cycle, iterative process using open-ended questions that explored barriers and facilitators to CR implementation during the pandemic. Interview notes were coded into themes using inductive thematic analysis. Informed by the TDF and the CFIR, we then categorized each theme into behavioral (capability/motivation) and contextual (inner/outer setting) determinants, respectively, of clinic- and home-based CR implementation.

#### Step 1B. Contextual Inquiry

In parallel to conducting semi-structured interviews, we completed in-depth interviews with key clinician stakeholders at NYPH.^42,43^ We used purposive sampling to identify members with direct experience administering CR and/or similar programs that use telemedicine at NYPH. Each in-depth interview aimed to understand the CR workflow, user and contextual factors to CR implementation, patient population (e.g., sociodemographics, digital health literacy, etc.), and the use of telemedicine within the context of NYPH.

### Step 2 Methods: Design intervention prototype based on user and contextual factors

From May 2020 to March 2021, we engaged in an iterative series of design team prototyping workshops/meetings^44^ and journey mapping sessions^45,46^ to develop a nontraditional CR program prototype that fit the context of our racial/ethnic and socioeconomically diverse setting while optimizing feasibility, appropriateness and usefulness.^47^

Journey Mapping and design team prototyping workshops/meetings: First, we assembled a NYPH design team of CR clinicians (n = 3), digital health team members (n = 2), and a creative director with expertise in ImS and UCD (n = 1). Next, we engaged the design team in an iterative process of journey mapping and prototyping workshops to develop a nontraditional CR prototype that addressed the barriers and leveraged the facilitators from Step 1. The process began with visualizing key prototype design features (e.g., home-based telemonitoring and exercise equipment, educational videos, frequency of CR sessions), followed by journey mapping the patient experience (e.g., receiving the nontraditional CR program) and the CR clinician experience (e.g., administering the program) from the beginning to end of program participation. Journey maps were presented to design team members to stimulate engagement, deeper understanding of the challenges/opportunities, and discussion on how best to approach subsequent activities and design steps, while keeping the end users (i.e., patient and clinician) at the center of the design. In the case where follow-up from workshop sessions were needed, and to accommodate busy schedules, documented email threads were used to continue design feedback and prototype creation among various design team stakeholders. We continued the iterative process of journey mapping and design team sessions until we yielded prototypes of the following intervention components: hybrid CR program design elements (e.g., number of sessions, combination of clinic-based and home-based sessions, program components [exercise, education]), patient- and provider-facing home-based CR protocol (e.g., timing and details of full session, exercise modality/equipment, remote monitoring), and telehealth monitoring protocol/platform (e.g., how to navigate the telehealth platform).

### Step 3 Methods: Review and refine intervention prototype

From April 2021 to January 2022, we conducted a series of usability testing sessions with real-world CR clinicians and CR patients, followed by a final round of design team workshops and journey mapping to refine the intervention prototype.^48,49^ The goal of this step was to optimize usability and acceptability.

#### Step 3A. Usability Testing

Real-world usability testing of the initial prototype were conducted at a NYPH CR clinic located in the Washington Heights neighborhood of New York City.^49^ CR clinicians were included if they (1) were a full-time equivalent CR staff member, and (2) provided CR treatment to patients attending the NYPH CR clinic. CR patients were included if they (1) were enrolled in CR at the NYPH CR clinic as part of their standard of care secondary-prevention treatment, and (2) expressed interest in user-testing the nontraditional CR prototype. CR clinicians user-tested navigating the telehealth platform (e.g., identifying patient calendar, surveys, measurements, video call feature) to remotely administer (2-way video call) and monitor (heart rate and blood pressure measurements) patients during the nontraditional CR sessions. CR patients user-tested interacting with the telehealth devices (e.g., tablet, pulse oximeter and blood pressure cuff) to communicate/view the CR clinician (2-way video call) and take vital sign measurements (heart rate and blood pressure) before, during, and after aerobic and resistance exercise. Direct observations and field notes were used to document the patient-provider interaction and experiences with the equipment (e.g., telehealth devices, exercise equipment), telehealth platform, and protocol design/flow.

Step 3B. Archival Analysis, Design Workshops and Journey Mapping. After each usability session, the design team met to review what worked and did not work, and problem solved accordingly. Between design team workshops, archival analysis (e.g., characterize text from archived documents) of observation field notes, meeting minutes/notes, and emails were used to inform prototype and journey map revisions necessary to improve clinician- and patient-facing experiences.^50,51^ The archived documents were analyzed using thematic analysis and coded for factors that influenced patient and/or provider experiences related to equipment and the protocol (i.e., navigation, visibility, workflow). To ensure our themes aligned with UCD principles, we mapped each theme to key usability constructs (e.g., learnability, efficiency, memorability, error reduction, satisfaction, and exploit natural constraints).^26,52^ The usability themes guided the design team workshops and protocol refinement process, leading to ideal journey maps for each user experience (**Supplemental Fig. 1**).

## Results

From March 2020 to January 2022, we completed an iterative three-step UCD cycle that yielded a telehealth-enhanced hybrid CR (THCR) prototype that leveraged NYPH’s investment in Philips Healthcare’s remote patient monitoring (RPM) platform to optimize feasibility, acceptability, and appropriateness at the patient, clinician, and hospital system levels. Prototyping and user-testing ensured the model was both useful and usable by patients and providers. Below is an overview of the results from each step that led to the final THCR prototype.

### Step 1 Results: User and contextual factors of CR implementation

Step 1A. Clinic- and Home-Based CR Determinants. We contacted 10 key stakeholders at 3 geographically diverse, academic medical centers (New York, California, Michigan) and 9 agreed to participate in semi-structured interviews (67% female; CR supervisors/directors: n = 2 [physical therapist (PT), PhD researcher]; health system leaders: n = 2 [PT site director, doctor of medicine (MD) department chair]; clinician/staff: n = 5 [PT, registered nurse, exercise physiologist, patient navigator]). Details of stakeholders are provided in **Supplemental Table 2.** Determinants of CR implementation categorized by the CFIR and TDF domains are presented in Table 3.

For **clinic-based CR**, key contextual barriers (CFIR/TDF construct*[determinant theme(s)]*) to implementation included external policies (*social-distancing guidelines [e.g., “total volume of clinic-based CR patients will decrease due to social distancing”]*), patient needs and resources (*overwhelmed healthcare system [e.g., “(CR clinicians were) redeployed to inpatient side”]*), structural characteristics/available resources (*limited space/staff [e.g., “we don’t have much physical space”; “Per diem nurse started and she can’t go in clinic”]*), compatibility (*one-on-one sessions [e.g., “We can’t see people in groups anymore”*), and low relative priority (*non-essential service [e.g., “We temporarily closed on-site exercise and appointments”]*). Emotion (*patient fear [e.g., “patients were nervous to come in”; provider burnout [e.g., “small (staff) capacity and long wait list”]*) was a key behavioral barrier for clinic-based CR.

For **home-based CR**, key contextual barriers of implementation included external policies (*reimbursement [e.g., “As of now, this is a completely free service … because it cannot be reimbursed”, “(we are) delivering video visits for free”]*), available resources (*telehealth services/devices/exercise equipment [e.g., “(CR clinicians) aren’t offering any equipment- [they are] working with what the patients already have”; “The quality of video is highly dependent on the strength of (the patient’s) Wi-Fi signal”]), and compatibility (one-on-one sessions [e.g., “A lot of (electronic/telehealth) applications in the hospital are meant to be one-on-one”]*). Key behavioral barriers for home-based CR related to knowledge (*unfamiliarity with home-based CR/telemedicine [e.g., “(patients) have low health and technology literacy”*), beliefs about capabilities (*ability to administer home-based CR [e.g., “(clinicians) need to have better clinical skills to monitor remote patients”]*) and beliefs about consequences (*patient safety [e.g., “(people still ask) what is the safety of the service”*), and decision-making (*triaging patients [e.g., “how do you decipher which ones get it first?”]*).

A key **facilitator** for CR in general was the use of a hybrid delivery model because it addressed select barriers to clinic- and home-based CR. Key facilitators for home-based CR included collaborating with hospital administration/CR/telehealth champions/opinion leaders (*e.g., “Get buy-in from the leadership”*), leveraging existing CR workflow/electronic health record/telemedicine infrastructure and initiatives (*e.g., “[a home-based CR program] is consistent with the [hospital] goals to expand telemedicine and aligns well with the [hospital] telehealth initiative”*), and intervention adaptability (*e.g., “create as you go”*).

Step 1B. Local Contextual Factors. Two key clinician stakeholders (100% female; CR supervisor [PT], Associate Professor of Rehabilitation and Regenerative Medicine [MD]) agreed to complete a series of in-depth interviews to understand the local context of CR and telehealth at CUIMC/NYPH. The barriers and facilitators to clinic- and home-based CR from Step 1A were confirmed, with hybrid CR emerging as the ideal model to implement at NYPH (*e.g., the CR supervisor would “envision a hybrid type of program”*). When asked about telehealth and documenting remote visits, both stakeholders mentioned that all video visits and data collection happen in Epic (*e.g., “everything is in Epic”, “it’s all in the EMR”*) and that the staff are familiar with technology, but learning to adopt a new system may be challenging (*e.g., The good thing is that [the staff]is comfortable using the technology…logging onto MyChart isn’t unfamiliar, trying out a new way to do something is a barrier”*). Contextual inquiry also revealed that the primary CR patient population in the Washington Heights neighborhood of New York City were predominantly Hispanic, Spanish-speaking patients with varying levels of socioeconomic status and digital health literacy.

### Step 2 Results: Initial design of a telehealth-enhanced hybrid CR prototype

A total of 20 design intervention prototype workshops/meetings were conducted to design a nontraditional CR model that addressed the contextual (informed by CFIR) and behavioral (informed by TDF) determinants of CR implementation that emerged in Step 1, as well as offer the same core components of traditional CR. Of these sessions, 10 focused on hybrid CR programming (e.g., frequency of visits, exercise modality/equipment, education content/materials), 9 focused on telehealth (e.g., devices, platform, remote monitoring), and 5 focused on eliciting feedback from additional stakeholders (e.g., Hospital leadership/administration, NYPH telehealth working group). A total of 7 follow-up email threads among design team members and additional stakeholders were used to facilitate prototype design in between workshops/meetings.

The co-design process revealed that the nontraditional CR prototype should include the following **key design elements**: (1) combination of home- and clinic-based CR sessions (i.e., hybrid vs. clinic-based only), (2) fewer total number of CR sessions (i.e., 24 vs. 36), (3) reduce total duration of direct clinician supervision/monitoring (e.g., 20 mins vs. 60 mins of provider supervision via 2-way video call during home-based sessions), (4) real-time RPM of resting and exercise vitals (i.e., synchronous vs. asynchronous monitoring), (5) align with existing clinical workflow and telehealth infrastructure (vs. external processes/vendors), and (6) provide training for clinicians and patients. **Supplemental Table 3** outlines how each design element addressed Step 1 determinants (e.g., reimbursement, compatibility).

Throughout the design process, details of the prototype design evolved based on stakeholder feedback, NYPH infrastructure, and ability of the proposed design elements to address user and contextual determinants. For instance, the initial prototype leveraged Epic’s MyChart Video Visits, which aligned with existing clinical workflow and infrastructure, but required patients to have: (1) their own electronic device, (2) Wi-Fi, and (3) independently log into their Epic portal. Specifically, Epic’s MyChart Video Visits did not support interoperability between commercial patient monitoring devices (e.g., Fitbit, Polar HR monitor, store-bought blood pressure monitor) and the EHR, hindering the ability of real-time RPM during home-based sessions. Accordingly, we leveraged the expertise from our digital health team members and decided to partner with NYPH’s investment in Philips Healthcare’s RPM platform, which included: 1) freely available RPM devices (e.g., tablet, pulse oximeter, BP monitor and cuff) that wirelessly transmits BP and HR data to a web-based tracking database (eCareCoordinator [eCC]) during CR sessions/exercise; 2) Real-time integration of HR/BP data into the EHR (eCC interfaces with Epic); and 3) Telemonitoring-enabled CR-support via 2-way video calls. Moreover, to address key determinants highlighted by stakeholders in Step 1 (e.g., reimbursement, available resources), this platform was chosen to ensure patients had access to reimbursable resources (vs. out-of-pocket expenses) that support both cellular and Wi-Fi connectivity (vs. requiring Wi-Fi access and/or cellular data plan).

Ultimately, this process yielded a telehealth-enhanced hybrid CR prototype to offer 24, 60-minute CR sessions (with partial clinician supervision) over 12 weeks. The prototype combined home-based CR (e.g., remote exercise monitoring) with NYPH’s existing clinic-based CR (i.e., standard of care), EHR (i.e., Epic), and telemonitoring (e.g., Philips Healthcare RPM devices and eCC) infrastructure. Home-based exercise equipment included a stationary cycle-ergometer for aerobic exercise and ankle/wrist weights for strength training. This prototype was user-tested in Step 3.

### Step 3: Patient- and provider-level usability

We conducted 8 usability sessions (multiple sessions/patient) to simultaneously troubleshoot the patient (n = 3) and clinician (n = 2) experiences when using the eCC platform, RPM devices, home-based exercise equipment, as well as patient-clinician communication during the sessions. Since the end-users engage in more than one session throughout the program (i.e., 12-week program, 24 sessions), multiple usability sessions were conducted per patient (patient 1: 4 sessions, patient 2: 2 sessions, patient 3: 2 sessions; total sessions = 8). At least one clinician participated in each usability testing session. All sessions aligned with real-world clinical workflow (e.g., 60 minutes/session, scheduling, etc.). The NYPH design team completed intermittent design team workshops (n = 24) and journey mapping sessions (n = 3) to refine elements of the protocol.

Thematic analysis of the usability testing observations and meeting minutes revealed patient- and clinician-level themes (*codes*) for different prototype intervention components (Table 3). Patient-level themes for RPM devices were related to the ease of using the devices (*capability/comfort using the devices, visibility, navigation*) and technology disruptions (*Wi-Fi/Bluetooth connectivity*), while themes for exercise were related to comfort with ability to perform/use exercise modality/equipment (*capability/comfort, safety*) and flexibility with exercise experience (*adaptations/flexibility*). CR clinician-level themes for the eCC platform were ease of using the telehealth platform to remotely monitor patients (*visibility navigation*), technology disruptions (*Wi-Fi/Bluetooth connectivity*), and confidence in using the telehealth platform to safely monitor patients (*confidence in technology, safety concerns*). Each of these themes aligned with key usability constructs and principles (e.g., learnability, efficiency, satisfaction, etc.; **Supplemental Table 4**). Accordingly, design solutions were identified and incorporated into the refined prototype to improve the patient-facing experience with RPM and exercise (e.g., onboarding support, safety protocol, flexibility in programming based on patient progression) and clinician-facing experience with the eCC platform (e.g., revise eCC feature layout and interface, provide training aids/technical support, add safety check features to eCC protocol; Table 3). As for general programming, we detected positive comments and feedback on the patient experience when using the devices and process/workflow of the prototype. Changes made to the initial prototype based on Step 3 are outlined in **Supplemental Table 5**.

Table 4 outlines design elements of the final prototype after the entire three-step design process. The refined/final telehealth-enhanced hybrid CR prototype has been implemented at the NYPH CR clinic in the form of a NIH-funded pilot randomized controlled trial (UL1TR001873/KL2TR001874). Details of the pilot randomized controlled trial (RCT) have been registered on ClinicalTrials.gov (ID: NCT05328375). Briefly, we are conducting a single center, two-arm, 1:1 parallel group randomized pilot study comparing nontraditional CR with traditional CR among acute coronary syndrome (ACS) patients to evaluate the feasibility (e.g., recruitment, adherence) of conducting an adequately powered RCT. We are simultaneously assessing multi-level factors that influence the implementation of CR among post-ACS patients, as well as multi-level design feedback to inform another iterative UCD cycle before employing as a larger hybrid type I effectiveness-implementation trial.

## Discussion

The current study provides an outline of an iterative three-step process for applying UCD and ImS principles to identify user and contextual factors of CR implementation before engaging key stakeholders to co-design a theory-informed nontraditional CR program. The CFIR and TDF helped focus the identification of contextual-(outer setting, inner setting) and user-level (motivation, capability) implementation determinants, respectively, for general clinic- and home-based CR (Step 1). Design team workshops and journey mapping enhanced our ability to refine the nontraditional CR model based on usability themes that emerged from each patient and clinician-facing experience during usability testing. Collectively, this iterative process yielded a telehealth-enhanced hybrid CR model with the potential to optimize end-user experiences (e.g., usability, usefulness) and implementation potential (e.g., feasibility, acceptability, appropriateness) in real-world hospital settings serving racially and socioeconomically diverse populations.

Applying two complementary ImS frameworks (i.e., CFIR and TDF) to examine stakeholder perspectives on behavioral and contextual determinants of both home- and clinic-based CR implementation was an essential step to help inform the iterative co-design process. Although ample pre-pandemic research exists on clinic-based CR determinants (e.g., referral, transportation, work/family schedule, etc.),^53^ our theory-informed approach unveiled new pandemic-related barriers and facilitated simultaneous comparison of home vs. clinic based CR implementation determinants. While novel clinic-based CR barriers primarily included contextual factors linked to the inner (e.g., inability to conduct group-based sessions)/outer (e.g., social-distancing guidelines) settings, as well as stakeholder emotion (e.g., patient fear and provider burnout), key home-based CR barriers largely encompassed behavioral factors linked to motivation (e.g., beliefs about capabilities) and capability (e.g., decision making), as well as ongoing reimbursement constraints. The distinct determinants that emerged at different levels for clinic- and home-based CR highlight the need for design solutions that address both behavioral and contextual factors.

Despite these barriers, the pandemic presented unique contextual facilitators for home-based CR implementation. In October, 2020, CMS added CR to the list of approved telehealth services, partially addressing key reimbursement considerations.^13^ Nonetheless, this addition is temporary and coding limitations exist, highlighting the need to develop strategies to overcome reimbursement limitations, such as aligning with telehealth services that support reimbursable billing codes.^32,54^ This has been made increasingly possible by new hospital-wide telehealth initiatives and improved telehealth infrastructures. Accordingly, the use of a hybrid model that leveraged the hospital system’s telehealth infrastructure emerged as an optimal nontraditional design element from both system-level and provider-level stakeholders because it addressed select barriers to both clinic- and home-based CR models. The feasibility and effectiveness of the telehealth-enhanced hybrid CR model, as well multi-level determinants of implementation in the post-COVID-19 era, are currently being examined in the pilot RCT using a mixed-methods (e.g., surveys and semi-structured interviews) design.

A unique contribution of the current study is the combination of theory-informed ImS frameworks and usability testing methods to examine patient- and clinician-level experiences with an innovative *nontraditional CR model*. Although scientific calls to employ novel approaches to address the “research-practice gap” in cardiac rehabilitation,^13^ as well as comprehensive literature outlining best practices to combine and apply ImS and UCD exist,^25,26^ few studies have applied these concepts to the design and implementation of nontraditional CR models. Other studies have applied user-/human-centered design and/or theories to guide the development of nontraditional CR programs/apps and/or intervention elements (e.g., patient portal), but few have used these methods simultaneously and none have applied ImS principles to guide their design process. For instance, Joensson et al. (2019) performed a similar three step process that engaged multiple stakeholders to develop a theory-informed (self-determination theory) cardiac telerehabilitation web portal, called the ‘HeartPortal’, that provided design features to support patient-clinician communication and was found easy to navigate by heart failure patients.^55^ Similarly, Duff and colleagues (2018) engaged in a two-phase process to create a theory-informed exercise rehabilitation mobile app for adults with CVD. In phase I, they conducted a systematic review to identify behavior change techniques (BCTs), which informed the design of their app, followed by phase II, wherein they conducted focus group user testing and feasibility testing.^56^ Although both of these studies combined UCD and theory, their process solely engaged the patient stakeholder during product (e.g., patient portal, mHealth app) usability testing and design evaluation as opposed to eliciting feedback from other end-users, such as the healthcare professionals, and did not assess contextual factors using theory-informed frameworks that could influence implementation.

In contrast, Funahashi, Borgo, and Joshi (2019) applied a rigorous multi-level UCD approach that aligned with the hospital system needs to develop a technology enabled, evidence-based remote CR program;^57^ however, they did not infuse theory into the design of their program. To enhance rigor and replicability, behavior change theories and frameworks should guide the future design and development of complex CR interventions, as well as their implementation strategies. Interestingly, none of the aforementioned studies discussed and/or addressed reimbursement and most studies that employed UCD methods were outside of the U.S. (e.g.,

Ireland^56^, Finland^58^, Australia^59^, Denmark^55^), which may be a reflection of the different reimbursement policies and healthcare systems in which the programs were designed (e.g., private non-profit [Kaiser Permanente] vs. public, Europe vs. U.S. healthcare system). Moreover, few of these studies addressed racial/ethnic and socioeconomically diverse populations.

### Strengths and Limitations

This study has several strengths. First, this study expands the literature on the use of theory-informed implementation frameworks to characterize multi-level determinants of CR implementation to inform intervention development. Moreover, we provide an applied example of using the CFIR and TDF as complements to each other in the context of CR. The use of these frameworks provides a foundation to develop future multi-level implementation strategies. Second, this is among the first studies to combine both UCD and ImS methods at the early stages of nontraditional CR development.^23^ Third, the entire design process aligned with the clinical workflow and telehealth infrastructure of the hospital system. These findings, however, should be interpreted in the context of several limitations. First, patient stakeholders were not included on the design team, which may have influenced the design of the initial and refined prototype. To address this limitation, real-world CR patient stakeholders were included in the usability testing sessions (Step 3) and CR clinicians with >10-30 years of direct experience administering CR to our target patient population were included on the design team. Second, given the iterative QI nature of this study, the measurement of usability and implementation outcomes lacked formal assessment (e.g., Acceptability of Intervention Measure)^60^ hindering our ability to quantify whether the refined prototype improved these outcomes. Third, semi-structured interviews were not audio-recorded, limiting our ability to produce verbatim transcripts. To address these methodological concerns, rigorous quantitative and qualitative data on implementation determinants, implementation outcomes and usability outcomes are being collected and analyzed in the pilot RCT. Last, this is a small, single-center, cross-sectional study based on a quality improvement project in an urban academic medical center, which may limit the generalizability of our findings. These limitations notwithstanding, our findings shed light on determinants of CR and can inform the design of nontraditional CR programs and future selection of implementation strategies to increase the uptake of CR in the telemedicine era. Our findings suggest that future implementation efforts should center around nontraditional CR programs (e.g., hybrid, telehealth models) coupled with implementation solutions that address both behavioral and contextual barriers to clinic- and home-based delivery. As a next step, we will assess implementation outcomes and implementation determinants of the hybrid CR program at a NYPH CR clinic in the form of a NIH-funded pilot RCT.

### Future Directions

Additional research is needed to understand the optimal design by which a nontraditional CR model can improve patient and provider experiences and clinical outcomes in racial/ethnic and socioeconomically diverse settings, with the goal to mitigate inequities that exist in access to CR. Accordingly, future research should consider equity recommendations (e.g., focus on reach from the very beginning; use an equity lens for implementation outcomes) at the early stages of intervention development.^47^ Moreover, given the persistent dismal uptake of CR, future research should complement the traditional translational research pipeline with implementation science methods and frameworks integrated into early stages of intervention development.^22,23^ Our study provides an applied example, and our results will inform the feasibility, acceptability and effectiveness of THCR in a low socioeconomic status setting serving majority racial/ethnic minorities with Medicaid. The results of our mixed methods pilot study will inform the development of theory-informed implementation strategies for future multi-site studies.

## Conclusions

This paper provides an applied example for integrating user-centered design and implementation science principles into the early-stage development of a telehealth-enhanced hybrid CR model, while aligning with the behavioral and contextual factors of a real-world clinical setting. We found that prototyping and usability testing the provider and patient experience highlighted both generalizable and context-specific barriers, while also yielding meaningful design solutions. This process can serve as a model for future CR clinics that aim to design and implement a nontraditional CR program for their specific organizational/healthcare infrastructure and patient population, potentially maximizing our ability to reduce the evidence-to-practice gaps in CR implementation.

## Figures and Tables

**Table 1.** Overview of the data collection methods, stakeholders, deliverables, and outcomes considered during each step of the design process.

UCD Cycle	Methods/Considerations	Stakeholders	Deliverable(s)	Implementation and Usability Outcomes Considered
Step 1: Identify user and contextual factors	UCD: Contextual Inquiry ImS: Semi-structured Interviews CFIR (context) & TDF (user)	NYPH clinicians CR supervisors/directors Healthcare system leaders CR clinicians/staff	1. Multi-level determinants of CR (EBP) implementation (contextual and user)	Adoption
Step 2: Design intervention prototype	UCD: Design Team Workshops Journey Mapping ImS: Map design solutions to multi-level determinants Align design with real-world clinical setting and routine practice	CR clinicians Digital health members Creative director	1. Nontraditional CR Prototype (adapted EBP): (protocol and visuals) 2. Map of which multi-level determinants the nontraditional CR prototype addresses	Usefulness Feasibility Appropriateness
Step 3: Review and refine intervention prototype	UCD: Usability-testing Design Team Workshops Journey Mapping ImS: Observations Archival Analysis	Real-world CR patients CR clinicians Digital health members Creative director	1. Outline of design challenges and solutions 2. Refined nontraditional CR Prototype (protocol and visuals)	Usability Feasibility Acceptability

Notes: CR= cardiac rehabilitation, CFIR= consolidated framework of implementation research, EBP= evidence-based practice, ImS= implementation science, NYPH= New York Presbyterian Hospital, TDF= theoretical domains framework, UCD= user-centered design.

**Table 2.** Multi-level determinants of cardiac rehabilitation implementation during step 1 categorized by the Consolidated Framework of Implementation Research (CFIR) and Theoretical Domains Framework (TDF).

			Cardiac Rehabilitation Determinant Theme(s)	
Theoretical Framework	Domain	Construct	Clinic-Based	Home-based/Telehealth
CFIR	Outer Setting	External Policies	Social distancing guidelines	Reimbursement
		Patient Needs and Resources	Overwhelmed healthcare system	-
CFIR	Inner Setting	Relative Priority	Non-essential service, Provider redeployment	-
		Structural Characteristics	Limited number of staff	-
		Available Resources	Limited physical space and staff capacity	Limited staff capacity/hospital budget, Telehealth services/devices, Home-based exercise equipment/wifi access
		Compatibility	Inability to conduct group-based sessions	Inability to conduct group-based sessions, Technological issues
TDF	Motivation	Emotion	Patient discomfort/fear of in-hospital services, Provider burnout	-
	-	Beliefs about consequences	-	Patient safety
	-	Beliefs about capabilities	-	Ability to remotely monitor home-based sessions/use telehealth devices
TDF	Capability	Knowledge	-	Unfamiliarity with home-based CR/telemedicine
		Decision Making	-	Triaging patients

**Table 3.** Cardiac rehabilitation patient and clinician experiences during usability-testing and design solutions (Step 3).

Intervention Component (stakeholder)	Usability Themes	Codes & Examples	Design Solutions & Examples
RPM devices (Patient)	Ease of using the RPM devices	Capability, comfort *“Using the pulse oximeter while on the bike was hard”* Visibility *Patient struggled to see screen (of the tablet) while on the bike* Navigation *[the patient] didn’t know that pressing the Philips icon opened the tablet and didn’t know the PIN.* *[the patient] accidently pressed the mute button on tablet*	Onboarding Support *Provide binder with written instructions on how to use RPM devices* *In-person onboarding session to (1) introduce RPM devices, (2) demonstrate how to use RPM devices, and (3) have patient practice using the RPM devices*
	Technology disruptions	Wi-Fi/Cellular/Blue-tooth connectivity *The [pulse oximeter measurements] weren’t syncing. At this point [the patient] verbally reported [his heart rate]* *The [pulse oximeter measurements] weren’t populating in real-time. [the patient] removed it and then replaced it on his finger.* *[the patient] said “[the clinician] froze…”*	Alternative remote monitoring methods *Verbally report vitals via video call* *Manually enter vitals into tablet* Onboarding Support *Remote onboarding session to confirm RPM devices and features (e.g., video call audio, measurements, surveys) are working from home-based environment*
Exercise (Patient)	Comfort with ability to perform/use exercise modality and equipment	Capability, Comfort *“[the bike] feels different than the treadmill”* *“…exercise on the bike was harder, but in a good way.”* *[the clinician] spent 3-4 minutes of [the CR session] helping the patient adjust [the bike]* Safety *“[the patient] wants to make sure [the clinicians] could see him [while exercising on the bike]”* *“[the patient] needs to make sure he has something sturdy to hold onto [during strength training]”*	Onboarding Support *Provide binder with written instructions on how to setup and adjust the exercise equipment* *In-person onboarding session to (1) introduce exercise equipment and modality, (2) demonstrate how to use exercise equipment, and (3) have patient practice using the exercise equipment* Safety protocol *Provider patient with fully supervised remote sessions at the beginning of the program to ensure safety* *Provide patient with outline of safety protocol* Pre- and Post-exercise Surveys *Confirm patient location in the case of an emergency* *Confirm patient well-being* *Confirm exercise is completed*
	Flexibility with exercise experience	Adaptations/Flexibility *The clinician allowed a patient to try interval training on the bike. When asked “what did you like the most” the patient responded, “interval training”* *The clinician stated “…give flexibility based on patient needs”* *The clinician discussed “… importance of tailoring exercise to current energy levels.”*	Flexibility in programming based on exercise progression *The rating of perceived exertion target will vary week to week depending on the patient’s progression throughout the program* *The assigned weight (lbs) and number of repetitions per exercise will vary week to week depending on the patient’s progression throughout the program*
eCC platform (Clinician)	Ease of using the telehealth platform to remotely monitor patient	Visibility *During Video Visit: “it’s easy to view the patient with the eCC video call platform”* *When starting the Video Visit: “There’s no way to tell [in the eCC platform] that someone is waiting for you on the video call”* Navigation *When monitoring the patient: The clinician couldn’t toggle between the “video visit” tab and “trends” tab in the eCC platform.*	Revise eCC feature layout and interface *Split “trends” and “Video Call” feature on computer screen to allow simultaneous visibility of vitals and patient* Training *Provide CR clinicians with formal Philips Healthcare training on how to use eCC platform to enroll and monitor patients*
	Technology disruptions	Wi-Fi/Cellular/Blue-tooth connectivity *“..,syncing was an issue between pulse oximeter, cuff, and eCC platform…[clinician] had to enter information manually”* *“…there seemed to be a longer delay between audio and video… there may be an issue with Wi-Fi strength”* *“[the pulse oximeter reading] seems slower today… want to [verbally] read me the numbers”* *The clinician had to enter blood pressure measurement into eCC manually*	Technical support *Philips Healthcare representative to troubleshoot issues* Alternative remote monitoring methods *Ask patient to verbally report vitals via video call* *Manually enter vitals into eCC*
	Confidence in using telehealth platform to safely monitor patient	Confidence in technology *“The [blood pressure and pulse oximeter] readings [the clinicians] got were pretty accurate”* *“The BP seemed to work more accurately”* *“The pulse oximeter reading was fine…”* Safety concerns *Clinician instructed the patient that “more weight isn’t always better… we want to prevent injury”* *After the survey was completed- [the clinician] was waiting for the blood pressure measurement to come in [to the eCC platform], but it never did. The clinician said: “I have no idea what he’s doing”* *[Clinician] cannot see [the patients] feet to determine if wearing appropriate footwear.*	Add safety check features to eCC protocol *Surveys to confirm patient location* *Intervention rules to flag abnormal resting vitals* *Verbally confirm patient is wearing appropriate attire*
General Programming (Patient and Clinician)	Satisfaction	Positive comments/feedback The clinicians said that the patient “loved [the home-based model] and was ecstatic [with his experience].” The clinicians said that the patient “seem to like it… they like the equipment” The clinician said “all things considered, it was good” At the end of the user-testing sessions, the patient said “thank you again for everything and for the devices”	Satisfaction assessment *Administer satisfaction surveys on tablet as part of CR session assessment*
	Programming	Process/workflow (patient) *“[the patient] thinks the model works”* *The patient felt the “roll out was pretty smooth”* *“The process itself [the patient] really loved”* Process/workflow (clinician) *“…the pulse oximeter reading was fine and the dry run session was seamless”* *“(the session) was a bit of a learning curve with the patient- but they were happy to go through it”*	Kept foundational outline of measurements and surveys outlined in the home-based CR prototype *Sequence: resting vitals/surveys, aerobic exercise, strength training, post-exercise vitals/surveys*

Notes: CR= cardiac rehabilitation, eCC= eCareCoordinator, RPM= remote patient monitoring.

**Table 4.** Overview of the design elements included in the final nontraditional cardiac rehabilitation prototype.

Design Elements (User)	Clinic-Based Sessions	Home-based Sessions
Total Number of Program Sessions (Patient & Clinician)	**5**	**19**
Frequency of Sessions (Patient & Clinician)	**2 sessions the first week** **1 per month thereafter**	2/week
Duration of Sessions (Patient)	60 minutes	60 minutes
Duration of Monitoring Sessions (Clinician)	60 minutes	**60 minutes (first 2 sessions)** **20 minutes (each subsequent session)**
Patient Monitoring Platform, Mode of Supervision (Patient & Clinician)	Direct, In-person	**Remote, Philips eCC/Samsung Video Visits**
Patient Monitoring Devices (Patient)	Electrocardiogram machine, Blood Pressure Cuff and Monitor	**Pulse Oximeter, Blood Pressure Cuff and Monitor, Samsung Tablet**
Aerobic Exercise (Patient)	Modality: Walk/Jog Equipment: Treadmill	**Modality: Cycle** **Equipment: Stationary bike**
Strength Training (Patient)	Modality: Upper and Lower Body Exercises Equipment: Dumbbells	Modality: Upper and Lower Body Exercises Equipment: Ankle/Wrist weights
Training/Support (Clinician)	N/A	**Philips Training, Tech Support, and Cheat Sheets**
Training/Support (Patient)	Standard of Care	**2 onboarding sessions (1 in-clinic and 1 remote) prior to starting home-based sessions** **Onboarding binder w/ instructions**
Safety (Patient)	Standard of Care	**Verbally confirm patient location** **Surveys to confirm location & well-being**

Notes: eCC= eCareCoordinator. Bolded text indicate design elements of the initial prototype that were revised based on Step 3.

## Data Availability

The data that support the findings of this study are available in the Open Science Framework under the project title “Sex differences in the reward value of familiar mates in prairie voles” at https://osf.io/wmjfs/.

## Abbreviations

CFIR: Consolidated Framework for Implementation Research
CMS: Center for Medicare & Medicaid Services
CR: Cardiac rehabilitation
eCC: eCareCoordinator
HER: Electronic health record
UCD: User-centered Design
IS: Implementation Science
MD: Doctor of Medicine
NYPH: New York Presbyterian Hospital
PT: Physical Therapist
RCT: Randomized controlled trial
RPM: Remote patient monitoring
SQUIRE: Standards for Quality Improvement Reporting Excellence Implementation Studies
TDF: Theoretical Domains Framework
THCR: Telehealth-enhanced hybrid cardiac rehabilitation
